# Higher Admission D-Dimer Values Are Associated With an Increased Risk of Nonroutine Discharge in Neurosurgery Patients

**DOI:** 10.7759/cureus.9425

**Published:** 2020-07-27

**Authors:** Michael Karsy, Robert Kim, Mohammed Azab, Jonathan Harper, Jian Guan, Ilyas Eli, William Couldwell

**Affiliations:** 1 Neurosurgery, University of Utah, Salt Lake City, USA

**Keywords:** d-dimer, biomarker, outcomes, neurosurgery

## Abstract

Background

D-dimers are serum acute-phase proteins with a role in mediating inflammation that may be used as biomarkers for the prediction of deep vein thrombosis. Recent studies have shown that D-dimers can be used to predict prognosis and stratify risk in neurosurgical patients; however, a comparative analysis across diagnostic subtypes has yet to be performed.

Methods

A bioinformatics analysis evaluated neurosurgical patients with admission D-dimer levels between 2008 and 2017. Nonroutine disposition (e.g., skilled nursing facility, rehabilitation, other hospital, mortality) was primarily evaluated.

Results

A total of 1,854 patients (mean age 55.1±18.2 years, 55.4% male; mean admission D-dimer 4.83±7.78 μg/ml) were identified. Patient diagnoses included vascular (27.1%), trauma (16.4%), multiple diagnoses (15.7%), spine (15.6%), tumor (13.0%), and other (12.2%) causes. Univariate analysis showed that older age (p=0.0001), higher American Society of Anesthesiologists (ASA) score (p=0.0001), lower Glasgow Coma Scale (GCS) score (p=0.0001), diagnosis type (p=0.0001), longer length of stay (LOS) (p=0.0001), higher infection rate (p=0.0001), surgery in the past year (p=0.02), and higher D-dimer levels (3.4±4.9 vs. 5.4±8.7 μg/ml, p=0.0001) were associated with nonroutine disposition. Multivariate logistic regression showed that elevated D-dimers were independently associated with a greater relative risk of nonroutine disposition (relative risk [RR] 1.026, 95% CI 1.02-1.033, p=0.0001).

Conclusions

Elevated admission D-dimer values were independently associated with a 3% increased risk of nonroutine disposition per D-dimer unit after accounting for other factors. These results suggest that D-dimer values may help in stratifying patient risk models despite clinical heterogeneity. Further refinement of neurosurgical patient risk models using clinical variables and biomarkers may aid in resource allocation and early warning.

## Introduction

The use of biomarkers to predict neurosurgical patient prognosis remains an active area of interest because it may enable better targeted therapies and allocation of resources to patients, as well as guidance to patients and providers. D-dimers are fibrin degradation products released into the bloodstream after blood clot fibrinolysis that have classically been used for the evaluation of venous thromboembolism [1,2]. However, D-dimers are also serum acute-phase proteins (APP) that show upregulated expression after stress, infection, or worsening disease states. The recent literature has suggested that D-dimers can be used to evaluate and predict clinical prognosis in neurosurgical patients, including after subarachnoid hemorrhage [3-6], intracranial hemorrhage [7-9], ischemic stroke [10,11], and trauma [12-15] and in patients with dural arteriovenous fistula (dAVF) [16,17] and intracerebral [18,19] and spinal [20,21] neoplasms. However, within these studies, outcome measures are variable depending on the disease of interest, so a mix of elective and emergent patients are included and patients are derived from different institutions with variation in population demographics. A comparison of D-dimer biomarker prediction across different neurosurgical diseases, as one might come across in the average neurosurgical practice, has not been performed, limiting the ability to use this biomarker clinically. We aimed to explore the efficacy and accuracy with which D-dimers correlate with patient outcome.

## Materials and methods

Study sample

After the Institutional Review Board approval, we undertook a retrospective chart review using bioinformatic search parameters to evaluate patients admitted by the neurosurgery service from March 2008 to August 2017 after the initiation of a D-dimer protocol for deep vein thrombosis (DVT) detection at our institution, which required admission D-dimer levels on all patients. A total of 1,918 discrete patient encounters involving 1,854 patients were observed, where encounters involved separate admissions and discharges as previously reported [22]. D-dimer levels were acquired from blood samples (test #003057, reference range 0.0-0.4 μg/ml; ARUP, Salt Lake City, UT) at the date of admission. Other laboratory markers on the date of admission included white blood count (WBC), prothrombin time (PT), partial thromboplastin time (PTT), erythrocyte sedimentation rate (ESR), C-reactive protein (CRP), and procalcitonin.

Patient variables were collected from the medical record by chart review. Measurements of clinical severity included admission Glasgow Coma Scale (GCS) score and American Society of Anesthesiologists (ASA) score when documented. A diagnosis of culture-positive infection or treatment with antibiotics was marked as a patient with infection. DVT evaluation by ultrasound or CT angiography was noted. Major surgical procedures, defined as the need for use of the operating room under general anesthesia, were noted. Patients were allocated into diagnostic categories including vascular, spine, trauma, tumor, multiple, and "other diagnoses" based on the primary reason for admission. Tumor and spine patients were primarily elective patients, trauma patients were emergent admissions, and vascular patients were a mix of elective and emergent patients. The primary outcome for this study was discharge disposition. Routine disposition was defined as home or home health, whereas nonroutine disposition was defined as a skilled nursing facility (SNF) or long-term acute care (LTAC), acute rehabilitation, other hospital, or death.

Statistical analysis

For continuous and discrete variables, means with standard deviation and percentages were calculated, respectively. Continuous and discrete variables were analysed by t-test and chi-squared test, respectively. The nonparametric Mann-Whitney U test was used to evaluate median value differences. For logistic regression models, the outcome of nonroutine disposition was analysed. Univariate logistic regression was performed to calculated relative risks (RRs) and 95% CIs. Variables with a p<0.1 were entered into a multivariable model. A p-value <0.05 was considered significant. Statistics were analysed using SPSS Version 22.0 (IBM Corp., Armonk, NY).

## Results

Patient demographics

**Figure 1:**
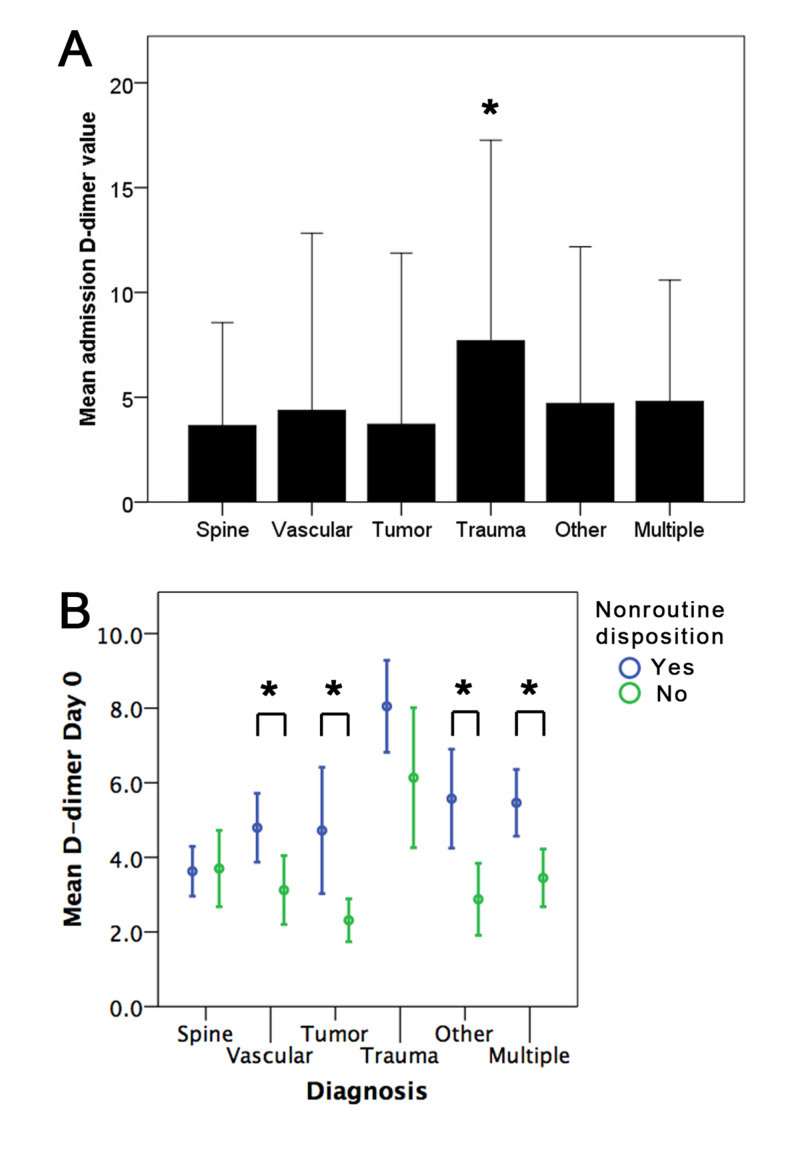
D-dimer associations with patient outcomes (A) Mean admission D-dimer values differed between trauma patients and the other categories (one-way analysis of variance, Tukey post-hoc, p=0.0001). (B) A significantly higher D-dimer level was seen for vascular (p=0.05), tumor (p=0.02), other (p=0.01), and multiple diagnosis (p=0.005) patients who had nonroutine discharge disposition. Spine and trauma patients did show differences in D-dimer levels for discharge dispositions. Error bars are 95% CI. *p<0.05.

A total of 1,918 patients (mean age 55.1±18.2 years; 55.4% male) were identified (Table 1). The most common diagnostic subtype was vascular (n=519, 27.1%), followed by trauma (n=315, 16.4%), multiple diagnoses (n=301, 15.7%), spine (n=299, 15.6%), tumor (n=250, 13.0%), and "other" (n=234, 12.2%). The mean length of stay (LOS) was 13.1±10.5 days, and most patients had a nonroutine disposition (n=1323, 69.0%). The majority of nonroutine disposition was to rehabilitation (n=813, 42.6%), followed by a SNF/LTAC (n=291, 15.2%). The average admission D-dimer level was 4.83±7.78 μg/ml. Mean D-dimer levels were significantly elevated and variable for trauma patients compared with the other diagnostic categories (one-way analysis of variance [ANOVA], Tukey post-hoc, p=0.0001) (Figure 1A).

**Table 1:** Baseline demographics from 1,918 neurosurgical patient admissions ASA, American Society of Anesthesiologists; GCS, Glasgow Coma Scale; DVT, deep vein thrombosis

Variable	Mean±SD or value (% total)
Age (years)	55.1±18.2
Sex (male)	1062 (55.4%)
Race	
Caucasian	1555 (81.1%)
Other	120 (6.3%)
Unknown	98 (5.1%)
American Indian	37 (1.9%)
Native Hawaiian/Pacific Islander	36 (1.9%)
Black/African	35 (1.8%)
Asian	37 (1.9%)
Ethnicity	
Non-Hispanic	1624 (84.7%)
Hispanic	136 (7.1%)
Unknown	158 (8.2%)
Median ASA score	3
Median GCS score	8
Diagnosis subtype	
Vascular	519 (27.1%)
Trauma	315 (16.4%)
Multiple	301 (15.7%)
Spine	299 (15.6%)
Tumor	250 (13.0%)
Other	234 (12.2%)
Length of stay (days)	13.1±10.5
Disposition	
Routine	415 (21.6%)
Nonroutine	1503 (78.4%)
Infection/antibiotics	704 (36.7%)
DVT treatment	428 (22.3%)
Major surgical procedure in previous 365 days	562 (29.3%)
Major surgical procedure within 120 days	683 (35.6%)
Admission D-dimer level (μg/ml)	4.83±7.78

Patient outcomes

 Nonroutine discharge disposition showed an association with admission D-dimer levels across diagnosis types, except for spine or trauma patients (one-way ANOVA, Tukey post-hoc, p<0.05) (Figure 1B). Univariate and multivariate logistic regression analysis compared clinical factors in patients with routine and nonroutine disposition (Table 2). Multivariate logistic regression demonstrated that age (RR=1.026, p=0.0001), trauma diagnosis (RR=1.54, p=0.04), greater LOS (RR=1.09, p=0.0001), infection (RR=1.5, p=0.001), D-dimer level (RR=1.04, p=0.001), and WBC (RR=1.02, p=0.05) were independently associated with a greater likelihood of nonroutine disposition. Non-Hispanic ethnicity (RR=0.64, p=0.04), higher GCS (RR=0.87, p=0.0001), tumor diagnosis (RR=0.6, p=0.004), and PTT (RR=0.984, p=0.02) were associated with lower rates of nonroutine disposition.

**Table 2:** Evaluation of association between clinical variables and discharge disposition ASA, American Society of Anesthesiologists; GCS, Glasgow Coma Scale ^a^Best fit model involved removal of sex as a variable. ^b^Colinear; GCS was removed from the multivariable model.

	Univariate		Multivariate	
	Unstandardized coefficients	p-value	Unstandardized coefficients	p-value
Admission D-dimer	0.06	0.006	0.2	0.05
Age	-0.2	0.0001	-0.3	0.009
Sex	0.04	0.07^a^		
Race	0.08	0.001	0.1	0.2
Admission GCS	-0.4	0.0001^b^		
Highest ASA score	0.3	0.0001^b^		
Treatment subtype	0.032	0.2	-0.08	0.8
Major surgical procedure within 120 days	0.08	0.03	-0.09	0.4
Disposition	0.09	0.0001	0.5	0.0001

## Discussion

Study findings

The results of this study suggest that higher admission D-dimer levels may be associated with worse patient disposition outcomes across various elective and emergent patients. This trend held for different categories of neurosurgical diagnoses, with the exception of trauma and spine, and after adjusting for various clinical risk factors such as DVT diagnosis, infection, and other elevated inflammatory markers. An approximately 3% greater likelihood of nonroutine disposition was seen for every unit increase in D-dimer levels on admission.

The implication for these findings is to stratify patient risk groups so that greater medical resources can be devoted earlier to high-risk patients. This may involve preemptive set-up for disposition, more aggressive physical therapy and rehabilitation, and discussion with family and staff regarding the expected longer treatment course for a high-risk patient. We did observe significant heterogeneity in D-dimer levels over this wide group of patients, likely as a reflection of the underlying physiology and disease differences. While it is premature to solely rely on D-dimers as a tool for prognostication, our data add to the existing body of literature for neurosurgical biomarkers and can be useful for the generation of DVT detection protocols at other institutions.

D-dimer as a disease biomarker in neurosurgery

D-dimers have previously been used to delineate outcome in a number of distinct diseases. In several small series, D-dimer levels in patients with subarachnoid hemorrhage or aneurysms have correlated with various outcome measures, including delayed cerebral ischemia, three-month Glasgow outcome scale, modified Rankin scale (mRS), and infections [3-6]. D-dimers have also been shown to correlate with poor outcomes in patients after ischemic stroke, including the Pediatric Stroke Outcome Measure and mRS [10,11], as well as thrombosis in dural arteriovenous fistulas [17]. Similarly, D-dimer levels correlate with worse outcomes in patients with traumatic brain injury (TBI), including posttraumatic cerebral infarction and hemorrhage [12,23-25], and with higher mortality in patients with gliomas or cranial or spinal metastatic disease [18-21,23,24,26].

Our study found a correlation of D-dimer levels and LOS with all diseases except for trauma. This was similar to only one other study, which found D-dimers within 24 hours were not correlated with the Glasgow Outcome Scale score after severe TBI [25]. However, in that study, the Glasgow Outcome Scale score did correlate with D-dimer after moderate TBI. Otherwise, we were able to show D-dimers could predict the increased risk of worsened disposition.

Acute-phase proteins

One reason D-dimers may be predictive of the outcome is because they serve as a biomarker of inflammation [27-29]. The APP response governs a cascade of pathological responses resulting in leukocytosis, elevation of acute reactive proteins (e.g., D-dimer, CRP, serum amyloid A, interleukins, tumor necrosis factor α), as well as clinical responses (e.g., pyrexia, hormonal alterations, muscle protein depletion). A typical response after a stressful event is the elevation of reactive proteins within 24-48 hours; however, the chronic inflammatory response may result from multiple events and may hinder physiologic recovery by potentially limiting tissue and wound healing, suppressing the immune system, and reducing physiologic reserve. This may likely increase susceptibility for patient complications and reduced mobility that can worsen disposition. Whereas several reactive proteins have well-defined specific clinical use (e.g., CRP and infection), the role of D-dimer remains to be better explored beyond a simple marker of thrombosis. All APPs show some non-specificity between infection and inflammation owing to the similar underlying molecular processes. Bridging the gap between serological laboratory changes and patient outcomes, which ultimately are complex and multifactorial, requires additional clinical stratification. D-dimer levels have not been predictive of prognosis for all neurosurgical diseases and it remains to be seen if this is simply the chosen study population or D-dimers serve as an epiphenomenon of inflammatory drivers that impact patient care [25,30].

Study limitations

One limitation involves the use of disposition as a common outcome. Although disposition is a complex end-point, impacted by disease severity, clinical treatment course, and socioeconomic factors, it nonetheless remains an important outcome related to treatment and cost. Disposition was comparable across different diseases and can be objectively verified by other researchers. Another limitation of this study involved the difficulty in accounting for disease severity across different disease groups. Adjusting patients for different disease severity was attempted using GCS, ASA score, disease subgroups, major surgical procedure, and presence of an infection or DVT. However, application of these variables differs among patients due to different standards in documentation for patient diseases. For example, GCS is not commonly acquired on our vascular patients as compared with a trauma patient. Further study of D-dimer biomarkers may benefit from replication of these findings and a prospective follow-up with additional clinical variables and outcome measurements. Although we did not specifically look at a D-dimer cutoff predicting a higher likelihood of nonroutine disposition, our prior studies did demonstrate that D-dimer levels of ≥2.5 μg/ml predicted a 30% higher likelihood of venous thromboembolism [18]. Future studies can potentially be helpful with generating cutoff values and clinical scores to predict outcome while using D-dimers.

## Conclusions

Higher admission D-dimer levels were independently associated with poorer discharge prognosis in a sample of neurosurgical patients, even after adjusting for disease severity and other clinical factors. A 3% greater relative risk of nonroutine disposition was seen for every one-unit increase in D-dimer levels. However, additional follow-up studies will be needed to objectively evaluate the ability of D-dimers to correlate with patient outcomes and improve predictive models. A better pathophysiological understanding of the inflammatory response in neurosurgical patients will also be necessary to progress from simply predicting outcome to intervening and improving treatments.
